# Visual analysis of commognitive conflict in collaborative problem solving in classrooms

**DOI:** 10.3389/fpsyg.2023.1216652

**Published:** 2023-12-20

**Authors:** Jijian Lu, Yuwei Zhang, Yangjie Li

**Affiliations:** ^1^Jinghengyi School of Education, Hangzhou Normal University, Hangzhou, China; ^2^Chinese Education Modernization Research Institute, Hangzhou Normal University, Hangzhou, China

**Keywords:** commognitive conflict, collaborative problem solving, cognitive diagnosis, visualization, learning-oriented feedback

## Abstract

In today’s knowledge-intensive and digital society, collaborative problem-solving (CPS) is considered a critical skill for students to develop. Moreover, international education research has embraced a new paradigm of communication-focused inquiry, and the commognitive theory helps enhance the understanding of CPS work. This paper aims to enhance the CPS skills by identifying, diagnosing, and visualizing commognitive conflicts during the CPS process, thereby fostering a learning-oriented innovative approach and even giving the script of technology-assisted feedback practices. Specifically, we utilized open-ended mathematical tasks and multi-camera video recordings to analyze the commognitive conflicts in CPS among 32 pairs, comprising 64 Year 7 students. After selecting the high-quality, medium-quality, and low-quality student pairs based on the SOLO theory, further investigations were made in the discourse diagnosis and visual analysis for the knowledge dimensions of commognitive conflict. Finally, it was discovered that there is a need to encourage students to focus on and resolve commognitive conflicts while providing timely feedback. Visual studies of commognitive conflict can empower AI-assisted teaching, and the intelligent diagnosis and visual analysis of CPS provide innovative solutions for teaching feedback.

## Introduction

In today’s knowledge-intensive and digital society, collaborative problem solving (CPS) has aroused increasing attention and is considered a critical skill for students to develop. It is defined as the capacity of an individual to effectively engage in a process whereby two or more agents attempt to solve a problem by sharing the understanding and effort required to come to a solution and pooling their knowledge, skills and efforts to reach that solution (OECD, 2015). Based on the PISA 2015 results, it was indeed found that Chinese learners had relatively lower performance in CPS compared to students from some other countries. It showed that students in Beijing, Shanghai, Guangdong, and Jiangsu and other places from China performed significantly worse in CPS than in math, science, and reading, ranking only tied for 25th place (51 countries or regions participated in the measure). The researchers discovered that the CPS task is less approachable for Chinese students after evaluating the CPS tasks in the worldwide assessment items (Zhou and Lu, 2017). As Chinese education system often focuses on standardized test, which may limit exposure to real-world problem solving and result in less development of CPS skills. However, it is worth noting that the efforts have been made to promote the students’ CPS skills in China. For example, the Chinese researchers had already positioned CPS measuring framework to evaluate the students’ development as soon as it was introduced in PISA 2015 (Wang, 2016).

The international assessment of CPS skills was initiated by the Assessment and Teaching of 21st Century Skills (ATC21S) project in 2008 (Yuan and Liu, 2016). After it, the Program for International Student Assessment (PISA), administered by OECD, introduced CPS as a component in its assessments. PISA 2015 marked the first large-scale assessment of CPS skills conducted within individual countries. Following PISA2015, Australia started a significant national-level evaluation (Li, 2017). The emphasis on CPS abilities in international assessments is due, on the one hand, to the significance of CPS skills and, on the other hand, to the social interaction view of individual mental development developed in recent years by sociocultural theorists, which provides theoretical and research case support for this aspect of the assessment.

In the study of CPS between pairs or groups, some scholars promote the development of new communication-oriented research paradigms based on the perspective of individual mental development and social interaction (Xu, 2018). Through communication, individuals in pairs or groups can share information, exchange ideas, negotiate solutions, and coordinate their efforts toward solving a problem. To better understand how social connections contribute to the development of personal mindfulness, communication-oriented research examines in-depth micro-behaviors within social interactions and communicative conversations. Professor Anna Sfard is a representative researcher in the new communication-orientation research paradigm. She put up the idea of commognitive, a theoretical presumption about how social interaction and individual cognition relates to one another: interpersonal communication and cognitive processes are essentially two sides of the same phenomenon (Sfard, 2007). Commognitive conflict refers to the cognitive conflicts that arise during CPS interactions among individuals. It occurs when participants in a collaborative setting encounter different perspectives, interpretations, or strategies while working together to solve a problem.

In order to promote CPS skills, it is an effective way to analyze and diagnose the commognitive conflict by observing the students work in pairs or groups. Visualizing the analysis of commognitive conflict during CPS allow educators to provide targeted feedback, better teaching intervention to students, thus promoting cooperative learning behavior. In the meantime, the development of artificial intelligence (AI) has made it possible to apply advanced statistical measures (e.g., RSM theory) to the practice of an online intelligent cognitive diagnostic system based on a test bank without difficulty. However, it still needs real classroom students’ commognitive conflict analysis theory to be the basis for the more complex commognitive conflict diagnosis and visualization.

To better develop students’ ability of CPS skills, our research was conducted to study the intelligent diagnosis and visual analysis of commognitive conflict. The research was conducted based on video feedback data obtained from a Sino-Australian collaborative project team. We analyzed the observed commognitive conflict within the knowledge dimension and classified them into conceptual, procedural, and contextual conflict, following the cognitive conflict structure proposed by Lee and Yi (2013). For conceptual knowledge, the sub-components include facts, conceptions, relations, and conceptual structure. These aspects pertain to understanding the fundamental principles, ideas, and relationships within a given subject area. Procedural knowledge encompasses thinking skills, ranging from simple to complex. These skills include description, selection, representation, inference, synthesis, and verification. Contextual knowledge focuses on specific contexts such as school, everyday life, and social/cultural/historical contexts. Understanding how knowledge is situated within different real-life situations allows for a more comprehensive and meaningful application of knowledge.

By identifying, diagnosing, and visualizing the commognitive conflict within knowledge dimensions during CPS, we can learn about students’ collaborative learning behaviors. This understanding promotes a learning-oriented innovative approach and even facilitates the creation of technology-assisted feedback practices. Moreover, the script of technology-assisted feedback practice can be to gain insights into the communication process, enable speech recognition for efficient feedback, and facilitate discourse diagnosis for improved instruction and learning outcomes.

Based on the background and purpose outlined above, the study focused on the identification, diagnosis, and visualization of commognitive conflict that arise during collaborative problem-solving (CPS) among student pairs. Firstly, we categorize the knowledge dimensions of commognitive conflict as conceptual, procedural, and contextual, so as to observe and analyze the commognitive conflict in students’ pairs. Secondly, three typical cases of high quality, medium quality, and low quality were selected through SOLO theory from 32 pairs of student peers for further case analysis. Finally, diagnosis and visual analysis of these cases are conducted to assist in cultivating students’ CPS abilities. We mainly study the following questions:

*Q1: What is the profile and visual diagnostic for the knowledge dimensions of commognitive conflict among student pairs?*
*Q2: How can the commognitive conflict be diagnosed in the discourse of student pairs?*
*Q3: How to visualize the commognitive conflict by 3D block diagram?*

By studying the students’ performance of commognitive conflict in CPS, it is possible to provide teachers with a theoretical framework and a visual case reference that enables them to provide innovative learning-oriented assessment and feedback practice in the classroom. It also gives guidelines and script materials for future speech recognition supported by artificial intelligence and commognitive conflict discourse diagnosis.

## Literature review

### The study of commognitive conflict

In recent years, the research on commognitive conflict has tended to extend in a broad sense, viewing commognitive conflict as a state produced by discrepancies between an individual’s cognitive structure and the environment or between various components within that structure (Lee et al., 2003). Commognitive conflicts, which are cognitive conflicts that arise during communication, exist in the communication of different vocabulary usage, rules of evidence, etc. (Sfard, 2008). From a cognitive perspective, the heterogeneity of a team’s knowledge gives rise to diverse cognitive conflicts, which, in turn, facilitates the activation of more flexible cognitive mechanisms. These mechanisms enable the fusion of divergent cognitive schemata, ultimately leading to the creation of new cognitive constructions (Perry-Smith and Shalley, 2014). As the communicative interaction between people with different knowledge structures generates new patterns of knowledge connection, creative friction or creative chaos emerges (Zhang and Ni, 2006), stimulating various types of information exchange and the discovery of new solutions.

Sociocultural theory academics’ research on the social interaction view of individual mental development has offered a theoretical and empirical basis for the assessment of commognitive conflict. Vygotsky (1962) originally proposed that learners acquire knowledge most effectively through interaction, dialog, and negotiation in social, authentic learning situations that promote the holistic development. This not only improves the competency and learning performance of the students, but also stimulates the cognitive growth of the group through cooperation and interaction. When students are confronted with socially authentic problem situations, they participate in the CPS process through interaction, dialog, negotiation, and other learning styles. At this time, members’ heterogeneous knowledge structures communicate with each other, and while they build complementary knowledge within the team, they also generate varying degrees of commognitive conflicts. And the proportion of commognitive conflicts in CPS was significantly higher than the traditional cooperative learning (Liang et al., 2017).

Sfard (2007) created the theory of commognition and categorized its levels and components based on various student-teacher and student–student communication dialogs. She developed the commognitive vision of mathematics as a type of discourse—as a defined form of communication, made distinct by its vocabulary, visual mediators, routines, and the narratives it produces. However, the theory has not yet codified the level of discourse and developed a more detailed description of the forms of conflicts, which is a challenging and innovative aspect of the study. Although commognitive theory has areas that need refinement, its applications are very broad and can serve as an effective research lens for different fields, and its potential has not yet been fully explored (Presmeg, 2016).

Due to the widespread application of commognitive theory, which has also received considerable attention from academics, the theory has been refined in practice. Regarding knowledge constructs for commognitive conflict, Gyoungho (2007) proposed a structural map of knowledge and beliefs that points to the students’ cognitive conflict analysis. This serves as a reference for the classification of knowledge content for commognitive conflicts.

Therefore, this paper argues that the commognitive version of discourse can be classified into conceptual, procedural, and contextual knowledge dimensions. In this way, we would able to observe how students collaborate to solve problems through classroom and to record the commognitive conflicts that arise during the learning process of interaction, dialog, and negotiation among members. If students can effectively manage commognitive conflicts, they will be able to foster cognitive development at both individual and group level. Moreover, this ability will also enhance their critical thinking skills and creative abilities.

### The study of commognitive conflict in CPS

In terms of problem-solving research and practice, scholars have constructed mature models to study and understand the process of problem solving. These models provide frameworks and guidelines for approaching problems effectively. Polya (1973) proposed a problem-solving model consisted of four-step process, which emphasizes the importance of understanding the problem thoroughly, strategic thinking, and critical reflection and helps develop effective problem-solving skills. Subsequent scholars have adapted and expanded upon Polya’s problem-solving model to cater to various needs and situations (Yu, 2008; Cao et al., 2016; Wei, 2019). For example, Schoenfeld (1985) divided the paradigm of problem-solving into six phases: preparation, exploration, strategy formulation, execution, evaluation and inquiry. In the “Inquiry” module, commognitive conflict refers to the cognitive conflicts that students may encounter while engaging in an inquiry-based learning process. When learners encounter these conflicts, they are presented with opportunities for deeper understanding and critical thinking. However, these researches have primarily focused on individual student problem solving of closed problems as the primary case study, and none of the models directly address commognitive conflict. When students’ pairs or groups solve mathematical problems in open environments, the cognitive model of CPS will become more sophisticated, and major commognitive conflict will occur.

In a study involving students’ commognitive conflict processes, Lewis and Mayer (1987) constructed a model on the process of comparing problem comprehension, arguing that students have a preference for the order of information provided in a problem and prefer problems that are in the same order. When students do not agree on the relational terms in solving comparison problems and the required arithmetic operations, comprehension errors occur and commognitive conflict arises. This type of conflict due to students’ preference for the order of problems will most likely present explicit commognitive conflict during CPS.

In terms of discourse analysis of commognitive conflict in CPS, Barron (2000), on the other hand, focuses on group-level characteristics of CPS, providing targeted strategies for examining cooperative group learning and providing explanations for the variability in the outcomes of collaborative activities. By recording and coding the quality of communication in the study groups, the characteristics of group interaction, problem-solving goal congruence are analyzed and the groups are classified into high quality and low quality problem solving. Iiskala et al. (2011) explore how metacognition becomes a socially shared phenomenon in their study of conversational episodes and characteristics during collaborative mathematical problem solving among high achieving students’ pairs. These researches provide a powerful reference for the discourse analysis of commognitive conflict in CPS.

In the context of commognitive conflict visualization, Ding (2009) visualized the knowledge refinement in CPS work. The study used a behavioral sequential approach to map students’ pairs and personal knowledge refinement curves in CPS. This visualization study of commognitive conflict in CPS offers valuable insights and ideas.

Overall, the majority of research on commognitive conflict in CPS has focused on the cognitive processes of individuals in problem solving and discourse conflict, whereas the visualization of commognitive conflict content classification and discourse is lacking. Therefore, our research utilized the SOLO theory to select student pairs of high-quality, medium-quality, and low-quality, and conducted a comprehensive investigation into identifying, visualizing, and diagnosing the commognitive conflicts during CPS. The statistical profile, discourse diagnosis, and visual analysis of commognitive conflict in CPS enable teachers to gain a deeper understanding of how students encounter commognitive conflicts and how different quality student pairs approach problem-solving. As a result, this provides teachers with timely and targeted guidance to support their students effectively, thereby enhancing students’ CPS skills. Moreover, the process of discourse analysis and visualization also offers a learning-oriented and innovative approach, providing a script for technology-assisted feedback practices.

## Methods

### Research participants and cases

Year 7, which is the present focus of the international CPS assessment, was selected prior to Year 8 in consideration of the exploratory nature of the project. The research segment, problem tasks, and research environment are all mostly consistent with the Australian partner side. 32 student pairs, consisting of a total of 64 seventh-grade students from the LH middle school in an urban area of the TZ district in BJ city, with moderate educational quality, were selected as the sample for the CPS recordings.

At the same time, the student outcomes were evaluated using the SOLO (Structure of the Observed Learning Outcome) five-level classification evaluation method (Biggs and Collis, 1982), which took into account the characteristics of the open-ended mathematical and contextual problems used in the project. The SOLO theory classifies observable learning outcomes into five levels: prestructural, unistructural, multistructural, relational and extended abstract structure. This resulted in the selection of the typical cases with the high quality, medium quality and low quality outcomes, as shown in Table 1.

**Table 1:** Selection of different student pairs’ CPS cases through SOLO.

Case	Case demonstration	Case interpretation
High quality P14	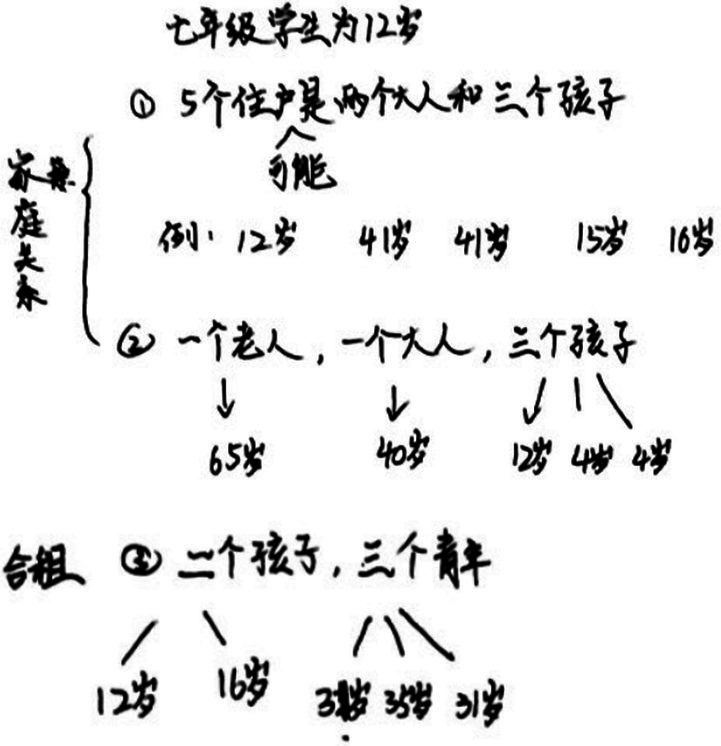	The conceptual and contextual knowledge was well presented, and several sets of correct and completed data were presented, both at the third level of the SOLO five-level evaluation. The presentation of hypothetical descriptions and family relationships for the five household roles, such as “possible” and “example,” is at the fourth level of SOLO Level 5 and is at the higher level of SOLO Level 4.
Medium quality P4	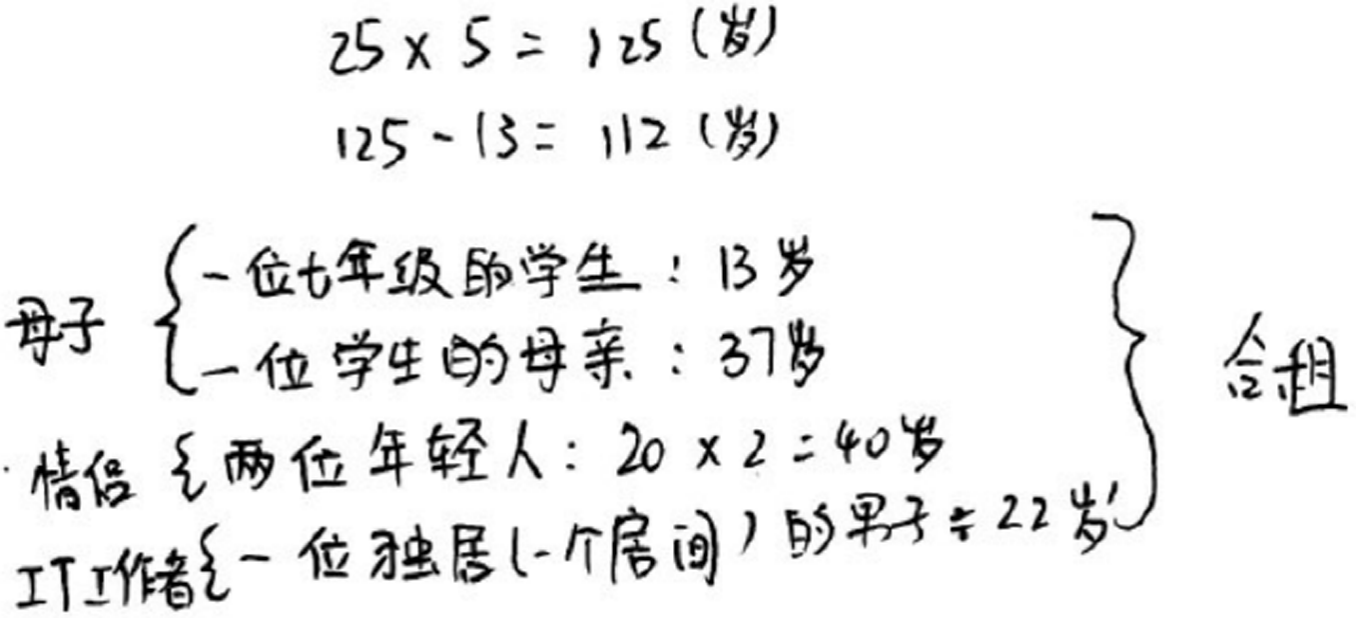	The conceptual and contextual knowledge was well presented, and a complete set of data was presented, reaching the second level of SOLO’s five-level evaluation. There are multiple assumptions about the internal relationships of the five resident roles, but the results of the thinking are incomplete and need to be supplemented.
Low quality P2	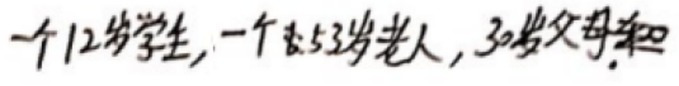	Only at the first level of the five levels of SOLO evaluation, and no accurate results were shown.

### Research task

The study utilized open-ended contextualized mathematical problems that better provoke commognitive conflict in communication (Clarke and Helme, 1998) from the Sino-Australian SEL project. The mathematical problem task is “Households and Age,” in which student pairs will collaborate to solve the problem, calculate the age of each person, and associate the social relationships of five people, as shown in Table 2. The student competencies examined in this task include the pedagogical problem-solving cycle involved in the International Institute for Frontier Mathematics Education, which promotes individual and group reflection on a dialectical cycle (Lu, 2017). Additionally, the study categorized the knowledge dimensions of commognitive conflict as conceptual, procedural, and contextual, and then examined and visually presented the features of the knowledge dimensions of the students’ pairs.

**Table 2:** Analysis of the “Household and Age” open-ended contextualized math problems.

Task content	Dimension	Description	Commognitive analysis
Household and AgeFive people live in a house; their total age is 25, and one of them is in the seventh grade. How old could each of the remaining four individuals be? What are the possible relationships between the five residents? Provide an explanation in a paragraph.	Conceptual knowledge	Factual, conceptual, relational, and conceptual structure disputes cause commognitive conflicts.	The overall age; The ages of each of the five persons; etc.
	Procedural knowledge	Thinking processes like description, selection, expression, reasoning, integration, and verification can lead to commognitive conflicts.	The calculation procedure of the ages of five people; etc.
	Contextual knowledge	Commognitive conflicts brought on by conditions in school, daily life, society, culture, and history.	Social relationships among 5 people; Roles of 5 people; etc.

### Research environment

**Figure 1:**
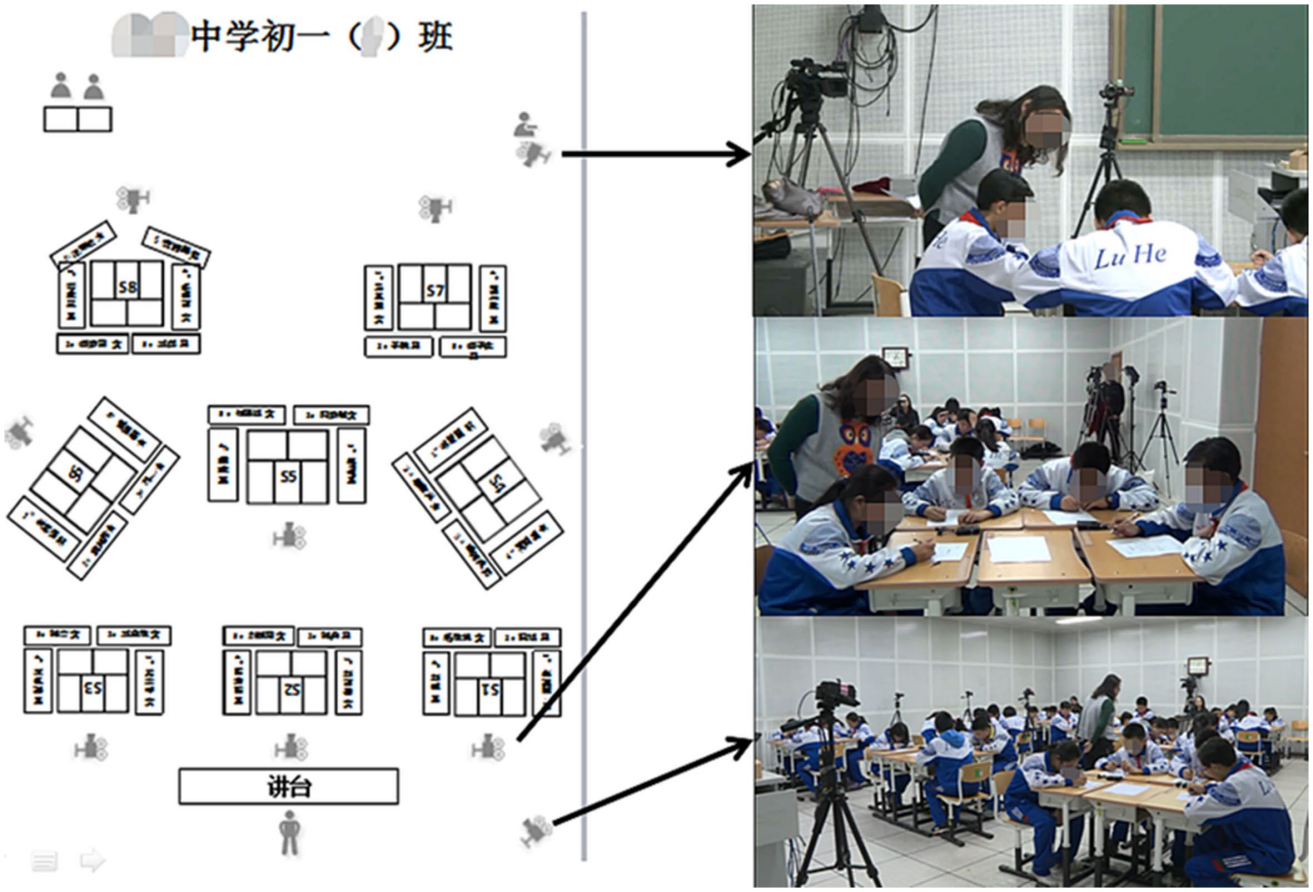
Classroom setup for data collecting for pairs math problem-solving projects.

Conforming to the specifications of the data collection classroom environment for the Sino-Australian Student Collaborative Mathematics Problem Solving Project, the study environment was built in a school-filmed classroom with which the children were quite familiar. Each group of 4–6 students in the videotaped classroom sat around a multi-tabled collocation table. The participants performed three tasks: individually, in pairs and in groups. In this paper, we only analyze the task conducted in pairs. A video camera was set up to capture the entire activity, and wireless microphones, such as left and right channels, were used to gather sound. Each group of students received pens, task sheets, rough draft paper, and other tools so they could complete the mathematical tasks. The study utilized 12 min of data from the problem-solving session involving pair participation. Figure 1 depicts the information in detail.

### Data analysis

During mathematical problem solving, student performance was videotaped, and the discourse was coded and analyzed. In this paper, we present a visual presentation and qualitative analysis of students’ performance in commognitive conflict during CPS. We analyze the differences in knowledge dimensions, the time and frequency of occurrences, as well as the diagnosis and visualization of commognitive conflict. The study coded the knowledge dimensions of commognitive conflict, identifying and classifying the types of conflict segments and recording the length of conflict for each segment. This resulted in statistics on the number, type, and average duration of conflict for 32 pairs of commognitive conflict groups. Two coders were utilized to confirm the validity of the coding results, and the consistency coefficient of the results was 0.913. Inconsistencies in coding were reviewed and deemed consistent by the coders.

To further analyze different quality student pairs’ commognitive conflict, the study used Nvivo12 software and 3D visualization block diagrams to visualize the data and thus present a more visual representation of commognitive conflict in CPS.

## Results

### Visual diagnostic of commognitive conflict knowledge dimensions

The study first encoded the commognitive conflicts of 32 student pairs and conducted an overall statistical analysis of the number of conflicts, average conflict duration, proportion of different types of conflicts, and resolution rate among student pairs. By coding and counting students’ pairs commognitive discourse, it was found that students’ commognitive conflicts were mainly concentrated in procedural and contextual knowledge, accounting for 47.5% and 46.50%, respectively. Conceptual knowledge accounted for only 6%. In terms of problem solving percentage, the problem solving percentage of procedural knowledge was 31%, and the solving rate was 65.3%; the contextual knowledge was 26%, and the solving rate was 55.9%. The percentage of problem solving based on conceptual knowledge was 6%, and the resolution rate was 100%. In terms of average conflict duration, the longest time was required to solve procedural knowledge, with an average of 52.77 s for one conflict. In contextual knowledge, the time for unresolved conflicts was 49.61 s, in which students were aware of the differences in their respective different mathematical contexts and therefore chose to postpone the conflicts. The details are shown in Table 3.

**Table 3:** Visual diagnostic profile of commognitive conflict.

Conflict type		Number and percentage of conflicts	Problem solving rate	Average duration of conflict (seconds)	Conflict type share
Conceptual knowledge	Solved	12(6.00%)	100%	15.45	6.00%
	Unsolved	0(0.00%)		0.00	
Procedural knowledge	Solved	62(31.00%)	65.3%	52.77	47.50%
	Unsolved	33(16.50%)		53.71	
Contextual knowledge	Solved	52(26.00%)	55.9%	50.38	46.50%
	Unsolved	41(20.50%)		49.61	

As it can be seen in Table 3, it is evident that the conceptual knowledge conflicts have the lowest percentage and the highest resolution rate, which indicates that students have a good grasp of the basic concepts of such problems.

To further analyze the commognitive conflicts among student pairs in CPS, the study selected high-quality, medium-quality, and low-quality case pairs using the SOLO theory. Visual analysis was then conducted on the quantity, occurrence, duration, and resolution status of commognitive conflicts in student pairs, as shown in Table 4.

**Table 4:** Diagnosis and visualization cases of commognitive conflict.

Pairs	The occurrence and duration of commognitive conflicts and whether they are resolved
High quality P14	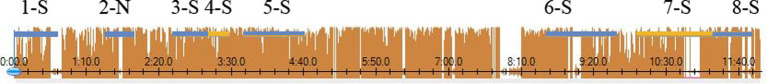
Medium quality P4	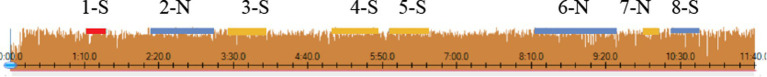
Low quality P2	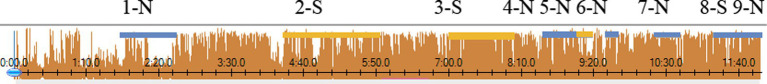

S in the table indicates “commognitive conflict was finally resolved,” while N indicates “commognitive conflict was finally not resolved.” The red line indicates conceptual knowledge conflict, the yellow line indicates procedure knowledge conflict, the blue line indicates contextual knowledge conflict. The timeline and commognitive conflict segment codes in the table were generated by Nvivo12 software.

Similarities and differences were discovered in the total number of occurrences, duration, and resolution status of commognitive conflicts among student pairs. The similarity lies in the total number of commognitive conflicts, with 8–9 conflicts occurring within a 12-min period. The difference is that each pair has its own characteristics in terms of the occurrence, duration, and resolution status of commognitive conflicts. The commognitive conflicts among high-quality student pairs emerged early and were resolved relatively quickly, with 7 out of 8 conflicts being resolved, while medium-quality pairs resolved 5 out of 8 and low-quality student pairs resolved only 3 out of 9, and the first two periods took longer. The information presented in the visualization diagram in Table 4 can also be expressed as the information shown in Table 5.

**Table 5:** Statistical comparison of different students’ pairs commognitive conflict.

Commognitive conflict classification	High quality	Medium quality	Low quality
	P14	P4	P2
Conceptual	/	1-S	/
Procedural	4-S, 7-S	3-S 4-S 5-S 7-N	2-S, 3-S, 6-N
Contextual	1-S, 2-N, 3-S, 5-S, 6-S, 8-S	2-N 6-N 8-S	1-N, 4-N, 5-N, 7-N, 8-S, 9-N

In terms of the categories of commognitive conflicts, different student pairs have fewer conflicts in the conceptual knowledge dimension. This is because the conceptual knowledge content of this mathematical task is not difficult, such as ‘the total age of five people, the age of seventh grade, and the age of the remaining four people’. When conflicts arise, students are more likely to resolve them. The commognitive conflicts mainly focus on the procedural and contextual dimensions, which is consistent with the conclusions obtained in Table 3. In high-quality student pairs, all procedural dimension conflicts have been resolved, while unresolved conflicts still exist in the middle and low-quality pairs. Therefore, the study will perform further discourse diagnosis and 3D block diagram visualization analysis on the procedural and contextual knowledge dimensions conflict, and reveal the characteristics and patterns associated with these conflicts.

### Discourse diagnosis of commognitive conflict

After selecting procedural dimensional commognitive conflict fragments, such as high quality P14-4S, medium quality P4-3S and low quality P2-2S and performing discourse visualization diagnosis, it was discovered that the procedural commognitive conflict is primarily manifested in the discourse of mathematical results after calculation errors by pairs. Table 6 takes the commognitive conflict fragments of the procedural knowledge dimension as an example and performs discourse diagnose from the beginning to the end of the conflict. This approach is also applicable throughout the entire study.

**Table 6:** Discourse diagnose of commognitive conflict in the procedural knowledge dimension.

Cases	Commognitive conflict in the procedural knowledge	Discourse diagnose
High quality P14-4S	[0:02:28.61] Girl 14A: Plus 12, that adds up to 34, and then counting those two.	14A Cause of conflict
	[0:02:53.58] Girl 14B: How did you add that? And then 34 plus who?	14B Found conflict
	[0:03:02.73] Girl 14A: With it plus it, it got to come out of it.	14A Explanation
		[0:03:06.11] Girl 14B: And then use it and subtract it.	14B Fixing conflict
	[0:03:08.49] Girl 14A: Right.	14A Affirmative response
	Medium quality P4-3S	[0:03:25.57] Girl 4A: If 13 years old, how much is left?	4A Cause of conflict
[0:03:32.58] Boy 4B: 122 …		4B Implicit conflict
[0:03:40.73] Girl 4A: Then the age of father and mother can be around 40 years old.		4A No conflict found
[0:03:55.11] Boy 4B: That’s 80 years old, plus 13, it’s 93 years old.		4B No conflict found
[0:04:10.49] Girl 4A: Then 122 minus 93, oh, it’s not right later. It’s 112 years old.		4A Found conflict
[0:04:20.09] Boy 4B: I see, I made a mistake in my calculations before.		4B Affirmative response
Low quality P2-2S	[0:03:51.95] Boy 2A: Add it up, the others add up to 107, minus that 13 year old, there has to be an old man. If you go by the fact that his dad and his mom are 40.	2A Cause of conflict
	[0:04:13.10] Girl 2B: Two four gets eight.	2B No conflict found
	[0:04:14.54] Boy 2A: Even if his parents are 40, two or four have to be 80’s. 67, exactly one old man.	2A Implicit conflict
	[0:04:30.33] Girl 2B: How did you calculate it, I’ll calculate it later ah.	2B Found conflict
	[0:04:33.24] Boy 2A: But that’s just one, two, three, four, just four people.	2A No conflicts found
	[0:04:36.68] Girl 2B: You started off wrong, 25 times 5 should get 125, you counted 120.	2B Explanation
	[0:05:10.65] Boy 2A: Subtract this kid, there’s 112 left, still counting two that what, two, one dad and one mom, that’s 67, 72.	2A Fix conflict

Discourse diagnosis helps the research to identify and analyze the linguistic, semantic, and interaction features of discourse, and reveal the underlying patterns and dynamics of how student pairs solve the procedural commognitive conflict. For example, in the high-quality student pairs, the discourse diagnosis reveals that Girl 14B had a commognitive conflict with Girl 14A’s calculation of 34 and further questioned the follow-up operation, which diagnosed as the cause and found of conflict. Girl 14B corrected the commognitive conflict after Girl 14A pointed to the draft document and made her explanations clear. She also suggested using 125 minus 34 and continuing to move backward, which was accepted by Girl 14A. The entire procedure, which took only 40 s, resolved the problem and even reversed some forward progress.

### Commognitive conflict visual diagnosis using 3D block diagram

The study selected different quality cases of contextual knowledge dimension commognitive conflict fragments and conducted 3D block diagram visualization analysis and diagnosis. This 3D block diagram is adapted from Lee’s structure of cognitive conflict. In research tasks, contextual knowledge is mainly divided into school mathematics knowledge and life experience. On the dimension of procedural knowledge, it is divided into arithmetic, mathematics quantitative certainty, and interval uncertainty. In terms of conceptual knowledge dimension, it involves basic mathematical concepts and other knowledge, consistent with the previous text. The path characteristics of commognitive conflicts can be intuitively seen from the 3D block diagram, as shown in Table 7.

**Table 7:** 3D block diagram of commognitive conflict in the contextual knowledge dimension.

3D block diagram	Commognitive conflict in the contextual knowledge dimension	Path
High quality: P14-5S	[0:03:53.23] 14B:A seventh grade student should be 12, right?	1-BA
	[0:04:02.55] 14A:Maybe 13.	
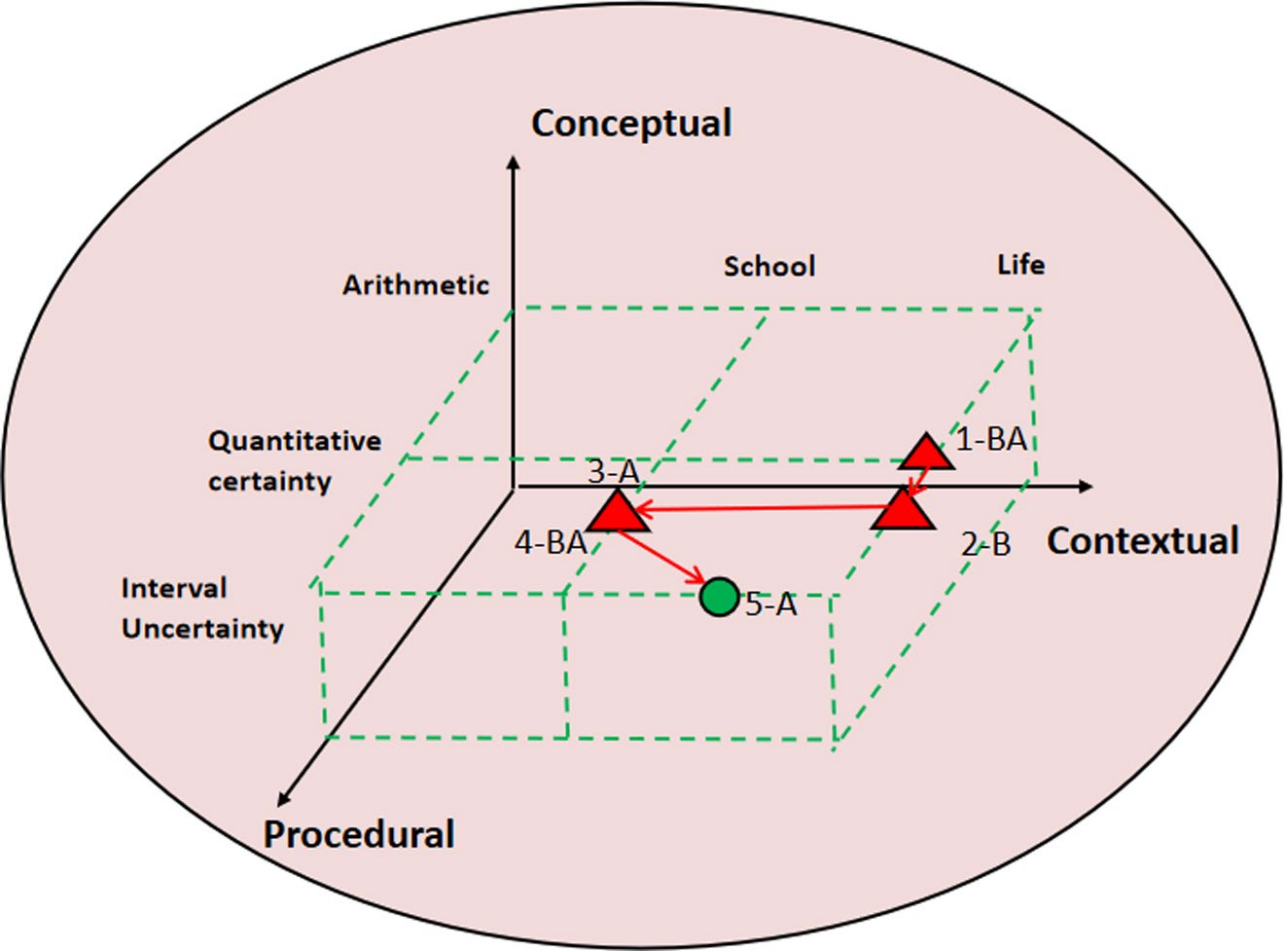	[0:04:15.31] 14B:If 13, parents are 41 years old. So it is 95, there are still 30 left. The remaining two might be an elder brother and sister.	2-B
	[0:04:25.39] 14A:An elder brother and an elder sister? Why not an elder brother and a younger sister?	3-A
	[0:04:35.45] 14B: If there is a younger sister, then the age gap between brother and parents would be too small.	4-BA
	[0:04:40.58] 14A:Alright.	
	14A:Considering the mathematical interval uncertainly, write “e.g.” in the paper.	5-A
Medium quality: P4-8S	[0:10:10.57] 4A:At the age of 13 in seventh grade, there are still 112 years left, and dividing 112 by 2 equals 56. Is that okay? Grandparents 56.	1-A
	[0:10:15.26] 4B:That’s too young. His father is 41 years old, how is it possible if grandparents only several years older.	2-B
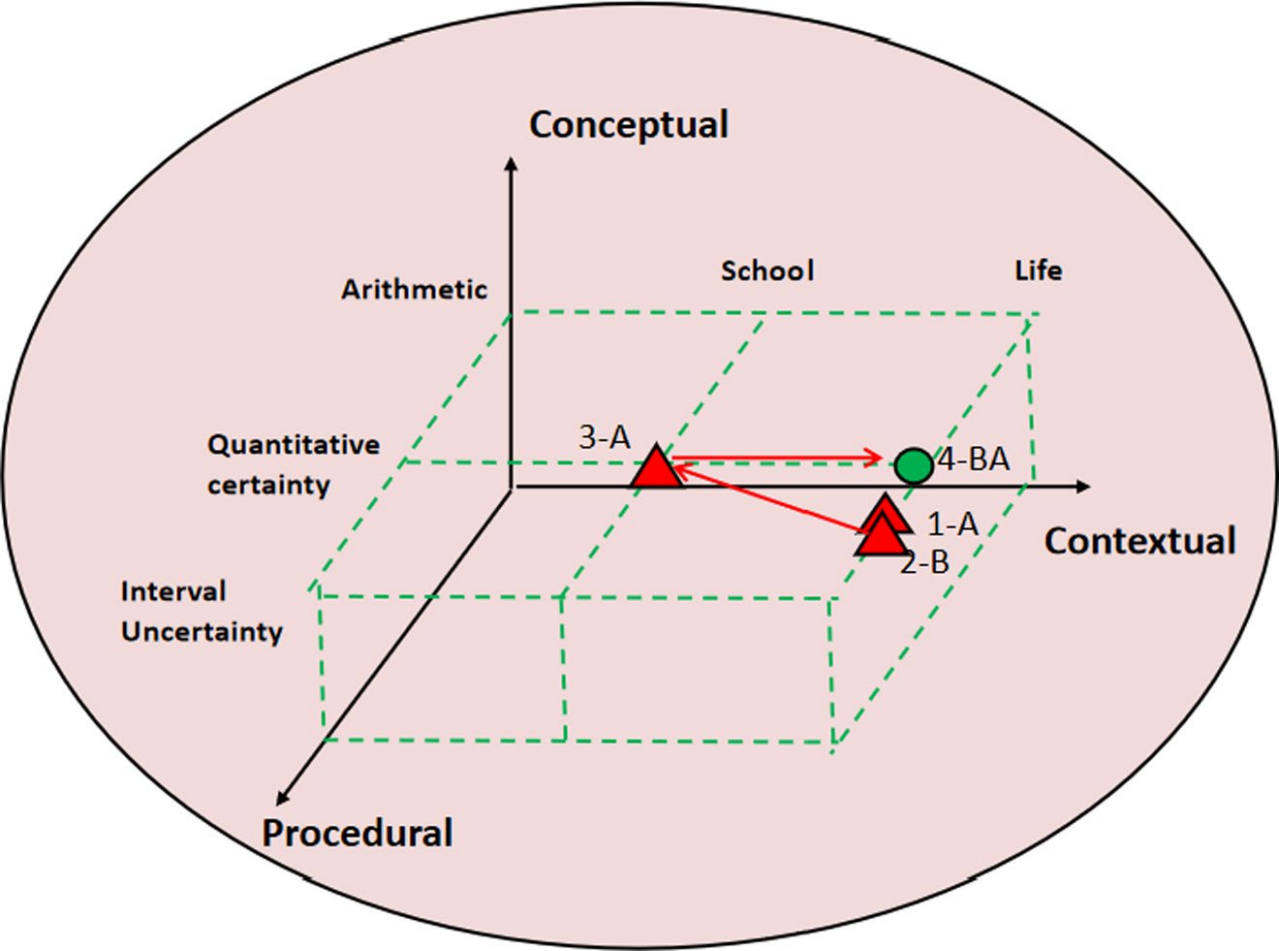	[0:10:25.37] 4A:Then there should be no grandparents, otherwise the total number would be too large.	3-A
	[0:10:30.65] 4B: Yes. So there should not be two elderly people.	4-BA
	[0:10:45.25] 4A:So, that should be a joint tenancy relationship.	
Low quality: P2-8S	[0:11:12.95] 2A:If there were a grandfather, it would be over 60.	1-AB
	[0:11:20.10] 2B:Then there’s an old man.	
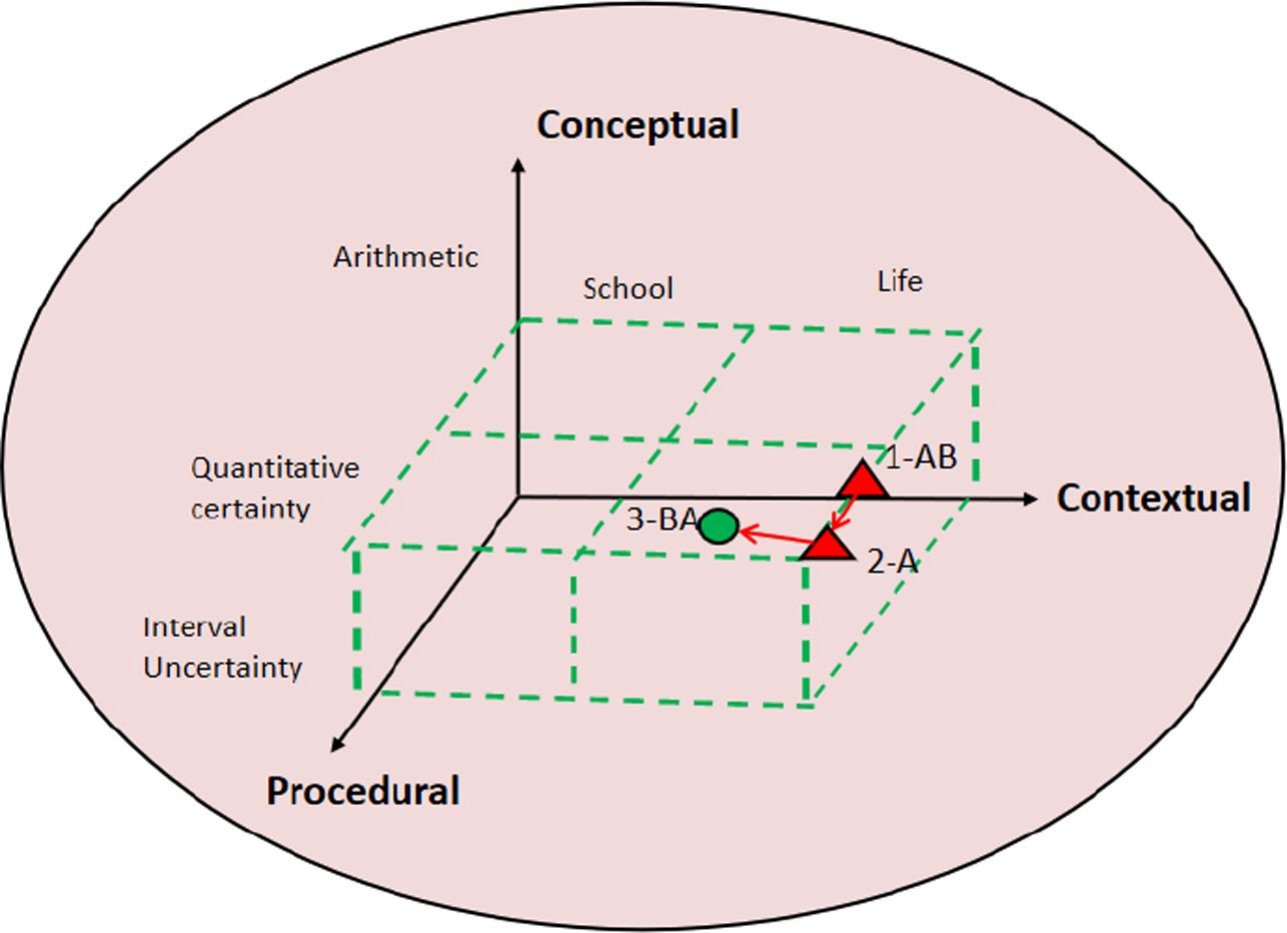	[0:11:25.54] 2A: If it’s 60, subtracting 60 from 123 will not work, as 60 is larger.	2-A
	[0:11:30.33] 2B:If 50?	3-BA
	[0:11:32.33] 2A:Try first. (Time is almost up, write 53 to try.)	

The 3D diagram allows for an intuitive visualization of the paths taken by student pairs in commognitive conflicts. Taking high-quality P14-5S fragments as an example, the conflict paths are as follows: 1-BA → 2-B → 3-A → 4-BA → 5-A. The specific process is described as follows:

1-BA represents student 14B, who based their judgment on life experience and considered the possibility that children are 12 years old. Meanwhile, student 14A revised 13 years old. 2-B indicates that student 14B, while considering the reasonableness of the gap between parents’ age and child’s age, concluded that the other two children should be siblings. 3-A indicates that student 14A experienced a communication cognitive conflict in response to the 2-B judgment made by student 14B. This conflict arose due to student 14A’s narrow focus on mathematical quantification in school mathematics. 4-BA represents student 14B’s explanation for their 2-B judgment. After student 14A confirmed the explanation, they performed calculations to determine the validity. 5-A indicates that when student 14A was recording the results, they considered the uncertain nature of mathematics and added descriptive elements such as “examples” in the column.

Research has revealed distinct path characteristics for commognitive conflicts among student pairs of different quality levels. In the case of the high-quality student pair (P15-5S), the process path for the emergence, negotiation, and resolution of commognitive conflicts follows a path of “life experience → school mathematics → life experience.” They also consider both the certainty and uncertainty aspects of mathematical problems. Similarly, the medium-quality student pair (P4-8S) follows a path of “life experience → school mathematics → life experience.” They take into account the age range in real life, which is then transformed into mathematical certainty, leading them to conclude that the five individuals have a shared rental agreement. Although their thinking is somewhat biased toward the life context, their logic remains reasonable. On the other hand, the low-quality student pair (P2-8S) only experiences the path of “life experience → school mathematics.” They fail to fully convert the discussed problems into the realm of school mathematics. Despite considering the interval uncertainty, their analysis lacks accuracy and depth.

In the 3D visualization diagnosis of commognitive conflict, the study mainly draws on Gyoungho Lee’s three-dimensional viewable framework (Lee and Yi, 2013); however, Lee’s related research mainly reveals static cognitive conflict and has not yet used the theory to analyze dynamic cooperative problem solving. This research uses the 3D analysis framework to present a dynamic path for commognitive conflicts and also refine the use of the analysis framework. Table 7 shows that the different quality commognitive conflict in CPS has a distinct pathway, the high- and medium-quality student pairs showing a “life experience-school mathematics-life experience” problem solving process. In this process, students make full use of their existing life experiences and mathematical knowledge, improve their own mathematical knowledge during the commognitive conflict with their pairs, and internalize and recreate based on their existing mathematical knowledge. This also coincides with Freudenthal’s theory of mathematics education—the idea of mathematical reality, mathematization and recreation (Freudenthal, 1973). It demonstrates that students may increase mathematics learning and create their own mathematical and life experiences by effectively resolving commognitive conflicts in CPS.

## Discussion

International assessments recognize CPS skills as critical for student growth, and commognitive conflict theory provides a theoretical foundation for individual knowledge creation and social engagement in collaborative challenges. Although established commognitive theories give a comprehensive description of conflict levels and elements, visualization studies of content classification and discourse levels are lacking. This research provides a visual representation of the knowledge dimension classification of commognitive conflict in CPS, as well as a discourse analysis of different quality student pairs. The Nvivo12 software was utilized in the study to visualize commognitive conflict with sound waves, as well as to present three-dimensional routes in a 3D analytic framework. This innovation presents commognitive conflict in a more concrete and visual manner. Specifically, intelligent diagnosis and visualization of student pairs’ CPS behavior can enable teachers to provide timely feedback, which provides new solutions for the observation of students’ engagement during CPS.

In this research, we choose the participates of pairs in the seventh grade, who have already learned addition, subtraction, multiplication, and division, were chosen to participate in the study. As open-ended contextualized mathematical life situations are added, and students focus on both arithmetic and character relationships, as well as each person’s age and total age. Table 1 presents the overall statistics of commognitive conflicts for the 32 student pairs. The researchers discovered a low percentage of conceptual knowledge conflicts, while the procedural and contextual knowledge commognitive conflicts were the primary challenges. Thus, the instructional interventions in these areas should be strengthened. Therefore, we conducted further investigations into commognitive conflicts in procedural and contextual knowledge dimensions through discourse diagnosis and visual analysis.

In the discourse diagnosis of commognitive conflict (Table 3), the study identified the origination, detection, interpretation, modification, and response of commognitive conflict. These specific linguistic features help us better understand when and where the commognitive conflict begins and ends, similar to the work done by Zhao et al. (2022). This analysis allows for a deeper understanding of the processes involved in commognitive conflict, enabling researchers and educators to effectively address and intervene in these conflicts to enhance student learning outcomes.

For visual analysis, Table 4 presents the visualization of commognitive conflicts using sound waves, displaying the occurrence, quantity, duration, and whether the conflicts are solved. Additionally, Table 7 utilizes 3D block diagrams to illustrate the path of commognitive conflict in the contextual knowledge dimension. This dynamic visualization shows how the conflicts occurred and possible resolutions. For example, in the case of the low-quality student pairs (P2-8S) mentioned in Table 7, if the teacher uses the visual diagram and identifies that these student pairs are experiencing difficulty in translating life experiences into school mathematics, timely intervention can be implemented. Recognizing this specific challenge allows the teacher to address it directly and provide targeted support or guidance to help these students overcome this hurdle.

To summarize, we diagnose and visualize the commognitive conflicts in student pairs’ CPS, which innovates the evaluation of learning-oriented feedback practices. Discourse diagnosis and visual analysis play a crucial role in enhancing the impact of feedback on student learning. As highlighted by Er et al. (2021), pairs’ feedback can be particularly effective in providing valuable solutions. As problem solving is a tool, a skill, and a process, the effective identification of commognitive conflicts is needed to improve CPS skills and even lead to creative solutions. However, due to limitations in research time and effort, further research is needed to analyze group work, employ additional visualization diagnoses, and explore the feedback of teachers on student cooperation issues, among other aspects. Addressing these shortcomings will be the focus of future research in this study.

## Conclusion

Based on the above studies, conclusions can be drawn from the need to encourage students to focus and resolve commognitive conflicts and make timely feedback; visualization studies of commognitive conflict can empower AI-assisted teaching as well as the intelligent diagnosis and visual analysis of CPS provide innovative solutions for teaching feedback.

### Encourage students to focus and resolve commognitive conflicts and make timely feedback

Existing commognitive theories give a detailed classification of commognitive conflict levels and elements, but research on content classification and visualization of discourse levels is lacking. In this study, we classify and visualize the knowledge dimensions of commognitive conflict in order to provide a theoretical and practical foundation for broader application. Future advancements can be made in the precise classification of commognitive conflict, the refinement of discourse analysis, and the development of visualization tools.

The visual analysis of the commognitive conflicts of different quality student pairs revealed significant differences in the duration, amount, and resolution of conflict, especially in the procedural and contextual knowledge dimensions. Thus, teachers need to encourage students to focus and resolve commognitive conflicts and make appropriate instructional interventions, which facilitate the development of students’ CPS from low to high quality. The commognitive conflict in CPS, when coupled with timely and targeted feedback, empowers teachers by fostering student engagement, deepening understanding, enabling personalized instruction, and so on. When students receive immediate responses to their contributions, it reinforces their engagement and participation. Furthermore, timely and personalized feedback provides guidance and clarifies misconceptions, helping students refine their understanding and address their unique challenges. Therefore, the dynamic coding and visualization of commognitive conflict in CPS can more effectively identify the types and manifestations of commognitive conflict in problem solving and thus provide a basis for teachers to provide targeted instructional interventions.

### Visual studies of commognitive conflict can empower AI-assisted teaching

During the research, we investigate explicit indicators of CPS, such as commognitive conflict categories, the occurrence and the average conflict duration etc., which are conducive to automatic identification and data analysis in the context of the rapid development of artificial intelligence (AI) for speech recognition. As AI technologies have the potential to reduce the workload for teachers and test developers (Yunjiu et al., 2022), their further development in the application of commognitive theory can provide directions and scripts for AI-assisted teaching, particularly in commognitive conflict discourse recognition and diagnosis in the future.

At the same time, computerized automated evaluation, at a time when AI development such as speech recognition is becoming more and more mature, has been able to begin to intelligently perform automated diagnostic analysis. Therefore, a more in-depth visualization study of commognitive conflict in CPS can provide ideas and references for future AI-empowered teaching and learning.

### Intelligent diagnosis and visual analysis of CPS provide innovative solutions for teaching feedback

In this paper, intelligent diagnosis and visual analysis are carried out according to the commognitive conflict of students’ pairs in CPS, which provides an innovative solution for visual presentation of teaching feedback. Visual studies of commognitive conflict can provide teachers with rich data that informs their decision-making. For example, when the visual diagram shows the low quality student pairs, by analyzing visual data, teachers gain insights into the quality of students’ interactions, and determine when and how to provide targeted instructional interventions. Its further development can provide analysis framework and case reference for teachers or even computer automation to evaluate students’ pairs problem solving level. This solution of intelligent diagnosis and visual analysis is intended to provide a deeper understanding of how students respond to feedback practices of commognitive conflicts in CPS in the future teaching, and to shift to more applicable results. This, in turn, promotes the development of individuals in the social interaction and communication. Therefore, in the future, it is necessary to strengthen research on the diagnosis and visualization of commognitive conflict in CPS which will provide scripts for future artificial intelligence, offer data support for targeted, timely, and personalized assistance.

## Data availability statement

The original contributions presented in the study are included in the article/supplementary material, further inquiries can be directed to the corresponding author.

## Ethics statement

The studies involving human participants are reviewed and approved by the Hangzhou Normal University Ethics Committee. Written informed consent to participate in this study was provided by the participants’ legal guardian/next of kin. Written informed consent was obtained from the individual(s), and minor(s)’ legal guardian/next of kin, for the publication of any potentially identifiable images or data included in this article.

## Author contributions

JL designed this study and drafted the original manuscript. YZ collected the data. JL, YZ, and YL had full access to the data and analysis. YL finalized the data analysis results. All authors contributed to the article and approved the submitted version.

## References

[ref1] Barron B. (2000). Achieving coordination in collaborative problem-solving groups. J. Learn. Sci. 9, 403–436. doi: 10.1207/S15327809JLS0904_2

[ref2] Biggs J. B. Collis K. F. (1982). Evaluating the quality of learning: The SOLO taxonomy (structure of the observed learning outcome). New York: Academic Press.

[ref3] Cao Y. Liu X. Guo K. (2016). Research on the Critcal level of the critical pedagogical Behaviors in middle school mathematics classroom. J Maths Educ 12, 73–78. doi: 10.14082/j.cnki.1673-1298.2016.04.011

[ref5] Clarke D. Helme S. (1998). Context as construction. Mathematics teaching from a constructivist point of view, 129–147.

[ref6] Ding N. (2009). Visualizing the sequential process of knowledge elaboration in computer-supported collaborative problem solving. Comput. Educ. 52, 509–519. doi: 10.1016/j.compedu.2008.10.009

[ref7] Er E. Dimitriadis Y. Gašević D. (2021). Collaborative peer feedback and learning analytics: theory-oriented design for supporting class-wide interventions. Assess. Eval. High. Educ. 46, 169–190. doi: 10.1080/02602938.2020.1764490

[ref8] Freudenthal H. (1973). Mathematics as an educational task. Dordrecht, Holland: D.Reidel Publishing Company.

[ref9] Gyoungho L. E. E. (2007). Why do students have difficulties in learning physics?: toward a structural analysis of student difficulty via a framework of knowledge and belief. Origin New Phys 54, 284–295.

[ref10] Iiskala T. Vauras M. Lehtinen E. Salonen P. (2011). Socially shared metacognition of dyads of pupils in collaborative mathematical problem-solving processes. Learn. Instr. 21, 379–393. doi: 10.1016/j.learninstruc.2010.05.002

[ref11] Lee G. Kwon J. Park S.-S. Kim J.-W. Kwon H.-G. Park H.-K. (2003). Development of an instrument for measuring cognitive conflict in secondary-level science class. J. Res. Sci. Teach. 40, 585–603. doi: 10.1002/tea.10099

[ref12] Lee G. Yi J. (2013). Where cognitive conflict arises from?: the structure of creating cognitive conflict. Int. J. Sci. Math. Educ. 11, 601–623. doi: 10.1007/s10763-012-9356-x

[ref13] Lewis A. B. Mayer R. E. (1987). Students' miscomprehension of relational statements in arithmetic word problems. J. Educ. Psychol. 79, 363–371. doi: 10.1037/0022-0663.79.4.363

[ref14] Li Y. (2017). The research on the project of 'Collaborative problem-solving online Assessment' in Australia —— an analysis based on the 'Conceptual assessment Framework' of ECD model. Primary and Secondary Schooling Abroad, 31–38.

[ref15] Liang Y. Zhu K. Zhao C. (2017). An empirical research on improving the depth of interaction through collaborative problem-solving learning activities. e-Educ Res 38:87-92+99. doi: 10.13811/j.cnki.eer.2017.10.014

[ref16] Lu J. (2017). Progress and trend of the research of international mathematics curriculum and teaching based on PME40. J Maths Educ 26, 77–81.

[ref17] OECD. (2015). PISA 2015 RELEASED field trial cognitive items. OECD Publishing. Available at: https://cnki.com.cn/Article/CJFDTOTAL-SXYB201705015.htm

[ref18] Perry-Smith J. E. Shalley C. E. (2014). A social composition view of team creativity: the role of member nationality-heterogeneous ties outside of the team. Organ. Sci. 25, 1434–1452. doi: 10.1287/orsc.2014.0912

[ref19] Polya G. (1973). How to solve it. Princeton, NJ: Princeton university press.

[ref20] Presmeg N. (2016). Commognition as a lens for research. Educ. Stud. Math. 91, 423–430. doi: 10.1007/s10649-015-9676-1

[ref21] Schoenfeld A. H. (1985). Mathematical problem solving. New York, NY: Academice Press.

[ref22] Sfard A. (2007). When the rules of discourse change, but nobody tells you: making sense of mathematics learning from a commognitive standpoint. J. Learn. Sci. 16, 565–613. doi: 10.1080/10508400701525253

[ref23] Sfard A. (2008). Thinking as communicating: Human development, the growth of discourses, and mathematizing. Cambridge, UK: Cambridge university press.

[ref24] Vygotsky L. (1962). Thought and language. Cambridge, MA: MIT Press.

[ref25] Wang L. (2016). Accessment and evaluation of key competences for student development-lessons from collaborative problem solving of PISA2015. Glob Educ 45, 24–30.

[ref26] Wei X. (2019). Problem solving and cognitive simulation—the example of mathematical problems. Beijing, China: China Social Sciences Press.

[ref27] Xu Z. (2018). 'Communicational Approach' to the study of mathematics education: background, hypothesis and conceptual framework. Stud For Educ 45, 98–110.

[ref28] Yu P. (2008). Theory of CPFS frame in mathematics learning. Nanning, China: Guangxi Education Press.

[ref29] Yuan J. Liu H. (2016). The measurement of collaborative problem solving: Analyzing of the measuring principle of PISA2015 and ATC21S. Stud For Educ 43, 45–56.

[ref30] Yunjiu L. Wei W. Zheng Y. (2022). Artificial intelligence-generated and human expert-designed vocabulary tests: a comparative study. SAGE Open 12:21582440221082130. doi: 10.1177/21582440221082130

[ref31] Zhang G. Ni X. (2006). Knowledge conflict process: a case study. R&D Manage 18, 66–73.

[ref32] Zhao J. Song T. Song X. Bai Y. (2022). Analysis on the linguistic features of conflict discourse in mathematical cooperation problem solving in China [original research]. Front. Psychol. 13:945909. doi: 10.3389/fpsyg.2022.945909, PMID: 36204754 PMC9531027

[ref33] Zhou J. Lu J. (2017). Collaborative problem solving task of PISA2015 and ATC21s and its enlightement to mathematics education. Maths Teaching in Middle Schools (in Chinese), 64–66.

